# Prevention and Management of Neonatal Hypothermia in Rural Zambia

**DOI:** 10.1371/journal.pone.0092006

**Published:** 2014-04-08

**Authors:** Karsten Lunze, Kojo Yeboah-Antwi, David R. Marsh, Sarah Ngolofwana Kafwanda, Austen Musso, Katherine Semrau, Karen Z. Waltensperger, Davidson H. Hamer

**Affiliations:** 1 Department of Medicine, Boston University School of Medicine, Boston, Massachusetts, United States of America; 2 Center for Global Health & Development, Boston University, Boston, Massachusetts, United States of America; 3 Department of Global Health, Boston University School of Public Health, Boston, Massachusetts, United States of America; 4 Save the Children US, Westport, Connecticut, United States of America; 5 Tropical Disease Research Centre, Ndola, Zambia; 6 Zambia Center for Applied Health Research & Development, Lusaka, Zambia; University of Alabama at Birmingham, United States of America

## Abstract

**Background:**

Neonatal hypothermia is increasingly recognized as a risk factor for newborn survival. The World Health Organization recommends maintaining a warm chain and skin-to-skin care for thermoprotection of newborn children. Since little is known about practices related to newborn hypothermia in rural Africa, this study's goal was to characterize relevant practices, attitudes, and beliefs in rural Zambia.

**Methods and Findings:**

We conducted 14 focus group discussions with mothers and grandmothers and 31 in-depth interviews with community leaders and health officers in Lufwanyama District, a rural area in the Copperbelt Province, Zambia, enrolling a total of 171 participants. We analyzed data using domain analysis. In rural Lufwanyama, community members were aware of the danger of neonatal hypothermia. Caregivers' and health workers' knowledge of thermoprotective practices included birthplace warming, drying and wrapping of the newborn, delayed bathing, and immediate and exclusive breastfeeding. However, this warm chain was not consistently maintained in the first hours postpartum, when newborns are at greatest risk. Skin-to-skin care was not practiced in the study area. Having to assume household and agricultural labor responsibilities in the immediate postnatal period was a challenge for mothers to provide continuous thermal care to their newborns.

**Conclusions:**

Understanding and addressing community-based practices on hypothermia prevention and management might help improve newborn survival in resource-limited settings. Possible interventions include the implementation of skin-to-skin care in rural areas and the use of appropriate, low-cost newborn warmers to prevent hypothermia and support families in their provision of newborn thermal protection. Training family members to support mothers in the provision of thermoprotection for their newborns could facilitate these practices.

## Background

In Zambia, similar to many resource-limited countries, some progress has been achieved in reducing mortality of children under 5 years of age, but less progress has been made to increase survival of neonates, or infants under the age of 28 days [1]. Globally, neonatal deaths account for 41% of mortality in children under 5 years of age, a rate that has been increasing over recent years [2]. Nearly 3 million newborns are estimated to die every year [3]. Immediately after birth, an infant is at highest risk of dying. About 25–45% of deaths occur during the first 24 hours [4] and 75% during the first week [5]. . Neonatal hypothermia, defined as an abnormally low body temperature of under 36.5°C), is a risk factor for newborn survival in low and middle income countries, particularly when associated with preterm birth and severe infections [6]. Hypothermia increases the newborn's metabolic requirements and is associated with hypoglycemia, hypoxia, and ultimately severe infections and newborn mortality [7]. In a recent community-based study conducted in Sarlahi, Nepal, mortality increased by approximately 80% for every 1 degree Celsius decrease in body temperature [8].

Many neonatal deaths, particularly those related to severe infections and prematurity, are preventable with relatively easy interventions to keep babies warm [9]. The World Health Organization (WHO) proposes a “warm chain”, or a series of interlinked procedures to minimize the risk of hypothermia in newborns, which includes warming the delivery place, immediate drying, skin-to-skin care, early and exclusive breast-feeding to promote close warming contact with the mother and provide energy to generate heat, postponing bathing, appropriate clothing and bedding, and placing mother and baby together [10]. However, even seemingly simple strategies such as skin-to-skin care are not consistently practiced in resource-limited settings [11].

Data from facility-based studies in Africa indicate that neonatal hypothermia is very common even in warm climates, with incidence rates at hospitals in Zambia ranging from 44 to 69% and high fatality rates [12], [13]. However, newborn care practices in sub-Saharan Africa at the community level, and their potential impact on neonatal hypothermia, are poorly understood.

While hypothermia has long been recognized as a potential threat to newborn survival in resource-limited settings, it has not received sufficient attention [14]. In Zambia, a majority of the estimated 18,000 newborn deaths yearly are attributable to conditions associated with neonatal hypothermia, such as severe infections (25%) or complications from preterm birth (37%) [1]. Implementing context-appropriate interventions for reducing hypothermia among newborns and might reduce associated risks and adverse health outcomes [15] and address poor neonatal survival in settings such as rural Zambia.

A better appreciation of environmental and local behavioral factors, and traditional practices that place neonates at risk of hypothermia in resource-limited settings in sub-Saharan Africa might improve the design and implementation of interventions to prevent newborn deaths in the communities. Understanding perceived barriers to and potential facilitators for preventing and managing hypothermia is key in ensuring that seemingly simple interventions can be implemented. The objective of this qualitative study was to explore and understand practices and attitudes regarding newborn hypothermia among communities in Lufwanyama District, Zambia, a typical rural area with limited access to health care and a poor infrastructure.

## Methods

### Design

We conducted focus group discussions (FGD) and in-depth interviews (IDI) from April to November 2010.

### Setting

This study was conducted in Lufwanyama District in the Copperbelt Province of Zambia, a large, rural, undeveloped district formerly referred to as Ndola Rural with a population of 78,500 [16]. Lufwanyama District has little physical infrastructure, poorly maintained roads that are frequently impassible during the rainy season; a near complete absence of electricity except that produced locally by diesel generators; and no piped water or sewage. The district health office, located outside the district in the town of Kalulushi, 14 kilometers west of the mining center town of Kitwe, was responsible for 15 formal health facilities (11 health centers and 4 health posts) staffed exclusively by nurses, nurse midwives, and/or clinical officers. At the time of this study, there was a single physician at one of two mission hospitals (St. Mary's, St. Joseph's) serving the district. Neighborhood health committees (NHC) are nationally recognized community structures, composed of volunteers who collaborate with health facilities to address community needs. As a consequence of all of these factors, a high proportion of basic health services are provided through several categories of minimally trained community workers – trained traditional birth attendants (TBAs), trained community health workers (CHWs), male motivators, safe motherhood agents, family planning agents, disease surveillance agents, malaria agents, tuberculosis agents, HIV/AIDS agents, family planning agents, as well as untrained TBAs and other volunteers.

### Subjects and sampling

We convened six focus groups of mothers of children aged between 0 and 23 months and eight focus groups with grandmothers, all with 10 participants. Inclusion criterion was willingness and ability to share experiences with childbirth and newborn care practices. Exclusion criterion was lack of consent to participate in the study. We randomly selected health facilities (HF) (two health centers and two health posts) as the sampling frame for informants. In each sampling area, we asked our network of TBAs and CHWs to identify mothers and grandmothers for the FGDs from two communities, one less than 5 km from the HF (“near”) and the other more than 5 km away from the HF (“far”). For the IDIs, we selected two community leaders (such as chief advisors, church leaders, or members of neighborhood health committees), one of whom was female, and four local heath committee members (two from near and two from far communities) from each of the four selected HF catchment areas. A team of community mobilizers trained by Save the Children contacted potential participants, explained the study purpose, and, if the potential study participants expressed interest in taking part, agreed with them on a time and place for the FGD or IDI that was convenient for them. Focus group participants were offered a refreshment drink and the equivalent of USD 5. The majority of potential participants approached agreed to take part in the study. We also interviewed district or provincial level medical officers, who were sampled purposively.

### Ethics Statement

Ethical approval was obtained from the Boston University Institutional Review Board (BU/IRB) and the Tropical Diseases Research Centre (TDRC) Ethical Review Committee in Ndola, Zambia. Before the interview took place, on the day of the FGD or IDI, we introduced the study team and obtained written informed consent.

### Data Collection

The field study team consisted of four female data collectors (three nurses and a teacher, SK), who conducted the interviews in the participants' native language. All data collectors were Zambian nationals from various tribes, who were not part of the communities they collected data from, but who were familiar with life in rural Lufwanyama from their previous work as nurses and teacher. They were supervised by a male physician (KL, trained in pediatrics, public health, and medical anthropology) with a research focus on maternal and newborn health. All had prior training and experience with qualitative research, and had no relationship with study participants prior to commencement. In addition, data collectors were trained by KL and KY-A, and interview guides were piloted and refined during the training. We conducted FGDs and IDIs at quiet places in the community, each lasting 45 to 90 minutes, and we audio-recorded them in addition to taking written records. All FGDs and IDIs utilized the same semi-structured discussion guide to allow for open-ended responses. The guide did not specifically mention thermal care, but rather asked questions and probed about general neonatal care and post-delivery practices that might reflect the management and prevention of hypothermia in the community, such as the location of births and persons present, the definition of the newborn period, common practices for the newborn immediately after birth, actions for newborns who look smaller than usual, and newborn danger signs. In the FGD, respondents received a number as anonymous identifier and were referred to as respondents 1 through 10. During debriefings at the end of each day, the study team reviewed the audio and written records, discussed interview strategies and experiences, and assessed data saturation.

### Analysis

Interviewers transcribed all audio recordings verbatim, and translated those conducted in Bemba or Lamba into English. One of the authors (S.K.), a bilingual speaker, verified translation into written English transcripts.

We used Nvivo 9.0 software [17] to code and analyze qualitative data. For the content analysis, KL started with open coding of the text to formulate analytic codes, and agreed with second coders (AM, KS) as to which codes to include in the analysis. We coded corresponding to each of the first-level codes (descriptors of important components of the FGD and IDI), using focused coding, guided by a specific thematic issue. We compared codes using theoretical memos and techniques such as systematic comparison and far-out comparisons [18]. We then compared our codes and agreed to refine first-level codes, organizing them into several categories (delivery practices, newborn care, danger signs, care seeking, community needs) to identify higher-level codes, relationships among categories, and to ensure saturation of categories. SK provided feedback on the data and the analytic process.

In order to increase the scientific rigor of our qualitative approach we took, we report our findings following the COREQ [19] and RATS [20] guidelines.

## Results

A total of 140 participants, all mothers or grandmothers, participated in FGDs. In addition, we conducted 31 IDIs (13 community leaders, 16 health committee members, and two district or provincial level medical officers).

### Hypothermia risk awareness

Respondents commonly voiced concern about exposing a newborn to cold. Mothers, grandmothers (caregivers), and health workers all believe the baby should be kept warm to emulate the conditions and thermal environment in utero:

FGD 03 with mothers, respondent 2 (R2): *When the cord is cut, they wrap the baby properly. Sometimes it is windy, and for a baby who has just been delivered, that is not good. So they quickly cut the cord, wrap it* [the baby] in *warm clothes and put it on the bed, so that it is kept warm, because the womb where it is coming from is warm.*

FGD 02 mothers, R1: *A baby is not supposed to be in a cold place.* [Why not?] R2 *It is because it comes from the womb where it is warm, so even outside we need to keep it warm.* [What about in hot season?] R1 *It is the same; we also keep it in warm clothes.*

IDI 17 with NHC member: *The baby is supposed to be covered because it can catch cold, so by wrapping the baby it is protected, considering that the baby has just come from the womb which is warm.*

### Hypothermia preventive practices

#### Birthplace preparation

Consequently, particularly in cold season, families prepare and warm a birthplace, usually with a charcoal brazier, to prevent the baby from being exposed to cold.

FGD 12 grandmothers: [What are newborns particularly vulnerable to?] *R2 Cold. R6 We put a brazier to warm the place where the baby is sleeping. R1 We try to protect the newborn from cold.* [Why?] *R1 So that the baby doesn't get a cough.*

Protection from the cold is thought to prevent diseases that result from being exposed to wind and cold without protection. Some diseases are believed to be directly hypothermia related. For example, though not specific to newborns, *akalaso* [“result of being exposed to cold”] is a concept often mentioned in context with newborns being exposed to cold. The term *akalaso* is often translated as “pneumonia”. These grandmothers explain the concept of cold and *akalaso* in the newborn:

FGD 08 grandmothers: *R5 Like during this time, in cold season, we put a brazier with charcoal in that space.* [What of in hot season, what happens?] *R5 We don't use a brazier. R4 Ok, if it is in hot season like October, we try to close up the windows to avoid wind and we also clean the place where the delivery is taking place. R5 Yes it's important, because a newborn should not be exposed to coldness. The baby might catch a disease like akalaso.*

#### Drying and wrapping

In Lufwanyama, drying and wrapping are part of newborn routine care. Both caretakers and health workers emphasize the importance of keeping babies warm with this simple measure:

FGD 12 grandmothers: *R8 We cover the baby with warm clothes, and avoid wind from outside. R1 Also we try to protect the newborn from coughs, so we warm the place by putting the brazier and also wrapping the baby in warm clothes.*

IDI 13 Neighborhood health committee (NHC) vice chair: *[What are common practices for the newborn immediately after birth?] When the baby is born… just after birth they try by all means to wrap the baby in warm cloths so that it does not catch cold.*

In most cases, caretakers or birth attendants just use a piece of cloth or a chitenge (a traditional garment worn by women wrapped around the waist or over the head), if available at delivery, to dry and wrap the newborn. Sometimes, pregnant women are advised in advance to arrange for clothing to be available, as exposure to cold is believed to be potentially fatal:

IDI 02 chief advisor: *Every newborn baby should be wrapped in warm clothes to avoid cold on its body. Otherwise the child can die and that is why we are advised to buy warm clothes in advance before the time of delivery.*

### Other thermoprotective practices

#### Immediate and exclusive breastfeeding

Although respondents did not mention breastfeeding in the context of neonatal hypothermia, newborns are commonly breastfed in rural Lufwanyama. However, in consideration of the strain a delivery puts on a mother, breastfeeding is not consistently practiced immediately after birth:

FGD 03 mothers: *R9 After delivery, you take a bath then you can feed the baby. R1 When the baby starts crying, it can be fed. R4 With me, when I give birth, if it is at night, I start breastfeeding the following morning.* [Why do you do that?] *R4 All my babies don't cry, they just sleep, and I do experience severe abdominal pain after delivery so I just let them sleep until the morning.*

FGD 13 grandmothers: *R6 If you deliver at night, you will just sleep until the following day that is when you start breastfeeding. Some babies just don't feed there and then.*

#### Delayed bathing

Women and grandmothers recalled that newborns used to be bathed immediately after birth, but this is now usually deferred until the day after delivery. Even though it is not always practiced, education from trained TBAs has helped to propagate delayed bathing as a thermoprotective measure:

FGD 13 grandmothers: *R9 Babies are given a bath immediately after delivery. After the cord is cut, they first wrap the baby and boil water and then they bathe the baby. R5 No, what I have seen is that the TBA wipes the baby and leave it like that until the following day. The following morning, the TBA will boil water to bath both the baby and the mother. R1 The way in which TBAs work…… after delivery they don't allow that baby to be given a bath that same day. No, they wrap the baby and emphasize that the baby should not be given a bath.*

Infants born small or prematurely are recognized as needing more intense thermal protection, so that bathing is delayed for longer:

FGD 11 grandmothers: *R9 Long ago, they used to bathe the baby just after birth immediately. R6 Some are too small, premature babies; they don't get bathed. R10. I have seen that with my baby, who was too small. So the TBA told me not to bathe the baby, and the baby took two weeks without a bath.*

In general, premature and smaller babies are recognized as being at higher risk for health complications. Caretakers and health workers agreed that those infants need particular attention, especially thermoprotection, beyond delayed bathing:

IDI 17 NHC member: *When the baby is born prematurely, it will be kept in a warm place until it is strong enough.*

FGD 03 mothers: [Some newborns look smaller than average. Are there any particular or different actions you take to protect these babies?] *R10. When that baby is born like that…. Because the womb is warm, so for that child who is premature to survive, you should look for warm cloths and wrap the baby properly. And the room in which the baby will be kept should be warm. A charcoal brazier should be kept there to keep that room warm, just like the temperature in the womb.*

FGD 05 grandmothers: [Some newborn look smaller than average. Are there any particular or different actions you take to protect these babies?] *R10 Those babies are kept in the house only, warm the room, wrap them in warm clothes, avoid the cold and the wind, and that child will grow. R8 We buy charcoal to light the brazier, which warms the room where the baby is sleeping to avoid it catching the cold which can result in a loss of a baby. R7 No bathing of that baby. It is just wiped and kept warm.*

### Handling of mother and newborn

Immediately following the delivery of the newborn, the birth attendant (if present) focuses primarily on the mother. The mother is usually cleaned and taken care of, so that she gets the chance to rest and recover.

FGD 12 grandmothers: R2 *Ok, what happens is that after delivery, the mother is given a massage and a bath, then is dressed in clean clothes. Then the newborn is given to her.*

In the interim, the newborn is sometimes just put aside or laid on the floor, exposing it to environmental cold. In some instances, only after cleaning and caring for the mother, is the newborn taken care of, often placed next to the mother in the bed or on the mat where the mother rests.

FGD 12 grandmothers: [When is the umbilical cord cut, is it before the placenta or after the placenta delivery?] *R7 After the delivery of the placenta.* [So what happens if the placenta takes a long time to come out, where is the newborn placed?] *R8 The newborn is just placed aside a bit.*

FGD 02 mothers: [Where is the newborn placed before the placenta is delivered?] *R2,9,8 (agree) Just on the floor where the mother is.*

FGD 14 grandmothers: [Where is the newborn placed before and after the placenta is delivered?] *R7 We just put the newborn baby on the floor after you have spread a cloth. R3 I wrap the baby and put the baby on the bed and after the placenta [is out], I put the baby with the mother.*

A mother's ability to provide thermal care for the newborn over an extended time of the newborn period, as is necessary for premature babies, is often limited by her need to return to work in the house and the fields.

FGD 07 mothers: *R7 When a mother delivers she has to stay at home to rest for some time.* [How long is a mother confined?] *R5 One month, so that the mother and the baby get strong. R10 We cannot go up to one month. For some of us here at the village, there is a lot of work waiting for you. You have to fetch firewood, water and food. So, you can only be confined at home for one week.*

FGD 01 grandmothers: *R10 Mothers are confined for two weeks or even a month if she has somebody to look for food. But if she is alone, she just stays for a week and starts working. R9 Village life is hard, you cannot be confined for too long, otherwise you could die of hunger.*

### Improvised devices to protect premature infants

For smaller babies and those perceived as needing more protection, heated water bottles are commonly used to provide external warmth. Both caretakers' and health workers' narratives reflect that warming babies, particularly premature ones, with warm water bottles at times for weeks and months and feeding them expressed breast milk are believed to foster their growth and development:

IDI 15 church leader: *The premature babies in the village, they take them to big health centres. But if they can't go, then for these babies, they use hot water in plastic containers and cover the baby to make them and cover them properly with blankets.*

IDI 06 female NHC member: *If it's a premature, you wrap the baby in warm clothes. Some get empty 2.5 liter containers [and fill them with warm water]. Then you put the container closer to the baby while the baby is wrapped. Sometimes you even put a brazier in the room to keep it warm. You try to breast feed the baby. If he can't suck, then you press out the milk and feed the baby using a cup. Most of the time, we don't bathe a very small baby until it grows a bit.*

FGD 13 grandmothers: *R1 When a baby is born prematurely at 6 or 7 months, I should put that baby in warm cloths, then I boil water into two bottles… The charcoal brazier in the house should not be put out. The house should be warm, all the time. I will put the baby in between the two bottles until 9 months elapses. Changing water regularly. R6 Just to add on, the same TBA who is conducting the delivery will be the one to tell you what to do. Then you run to the hospital when the situation becomes worse. R9 Ah, us we just leave it like that…. If God wishes that it lives, then it will live. But if not, then it can die.*

FGD 11 GM: *[Some newborns look smaller than average, are there any particular or different actions you take to protect these babies?] R10 My baby was very small so the TBA used to come in the early mornings and encourage me to boil water and put them beside the baby where he/she used to sleep and when I want to feed her, she just tells or asks me to press in a small cup.*

## Discussion

This ethnographic, qualitative inquiry of hypothermia-related practices, attitudes, and beliefs in rural Lufwanyama, Zambia, revealed that community members and health workers are aware of the danger of neonatal hypothermia. Community members report practices such as birthplace warming, drying and wrapping of the newborn, delayed bathing, and immediate and exclusive breastfeeding, which all contribute to keeping newborns warm. However, the warm chain as recommended by the WHO as the standard of care was not consistently maintained during the first hours after delivery, when newborns are at greatest risk and thermoprotection is most essential. Community members in the study area were not familiar with skin-to-skin care and did not practice it. Many mothers in Lufwanyama have to assume household and agricultural labor responsibilities soon after delivery, which makes it difficult for them to provide continuous thermal care to their newborns.

### Current practices

In Lufwanyama, there was a general awareness among caregivers and health workers that exposure to cold places newborns at risk for adverse morbidity and mortality. In the past, knowledge and awareness of neonatal hypothermia were poor even among health providers, as suggested in studies conducted in India [21] and in a multinational study [22].

This study's participants perceived heating the birthplace as a critical practice to protect the newborn from cold, particularly in the cold season. These findings are consistent with community-based practices explored in a qualitative study in rural Ghana [23], where most informants knew that keeping babies warm is essential for their health, but where traditional beliefs led to delays in thermal care. In contrast, studies from Nepal reported that the birth place was heated in only slightly over half of the settings [24], often only after birth [25].

In Lufwanyama, newborns were reported to be dried and wrapped, which prevents heat loss from evaporation; bathing was delayed; and particular attention was paid to smaller and premature newborns who were at higher risk of hypothermia. In this study, delays in drying and wrapping were reported with late cutting of the cord, i.e., after delivery of the placenta, and with attention to the mother. In a study in Tanzania, the practice of bathing newborns immediately after delivery was shown to be motivated by concerns about ‘ritual pollution’ [26]. In Ghana, early bathing was linked to reducing body odor in later life, shaping the baby's head, and helping the baby to sleep and feel clean. Informants felt that changing bathing behaviors would be difficult, especially as babies were bathed early in facilities [23].

Qualitative studies conducted elsewhere indicate that high-risk home delivery and newborn care practices that lead to heat loss remained common in resource-limited settings both in rural and urban areas. Examples given in studies mostly from South Asia include insufficient heating of the birth place, placing the uncovered newborn on the ground or other cold surfaces, delayed wrapping and early bathing [15], [27], [28]. A study from Dhaka, Bangladesh explained that babies were typically bathed soon after birth to purify them from the birthing process [29]. In Nepal, less than half of newborns were wrapped within the first 10 minutes after birth, and almost all of them were bathed within minutes or hours after birth [24] [25].

### Emerging theory and opportunities to improve thermal care

**Figure 1:**
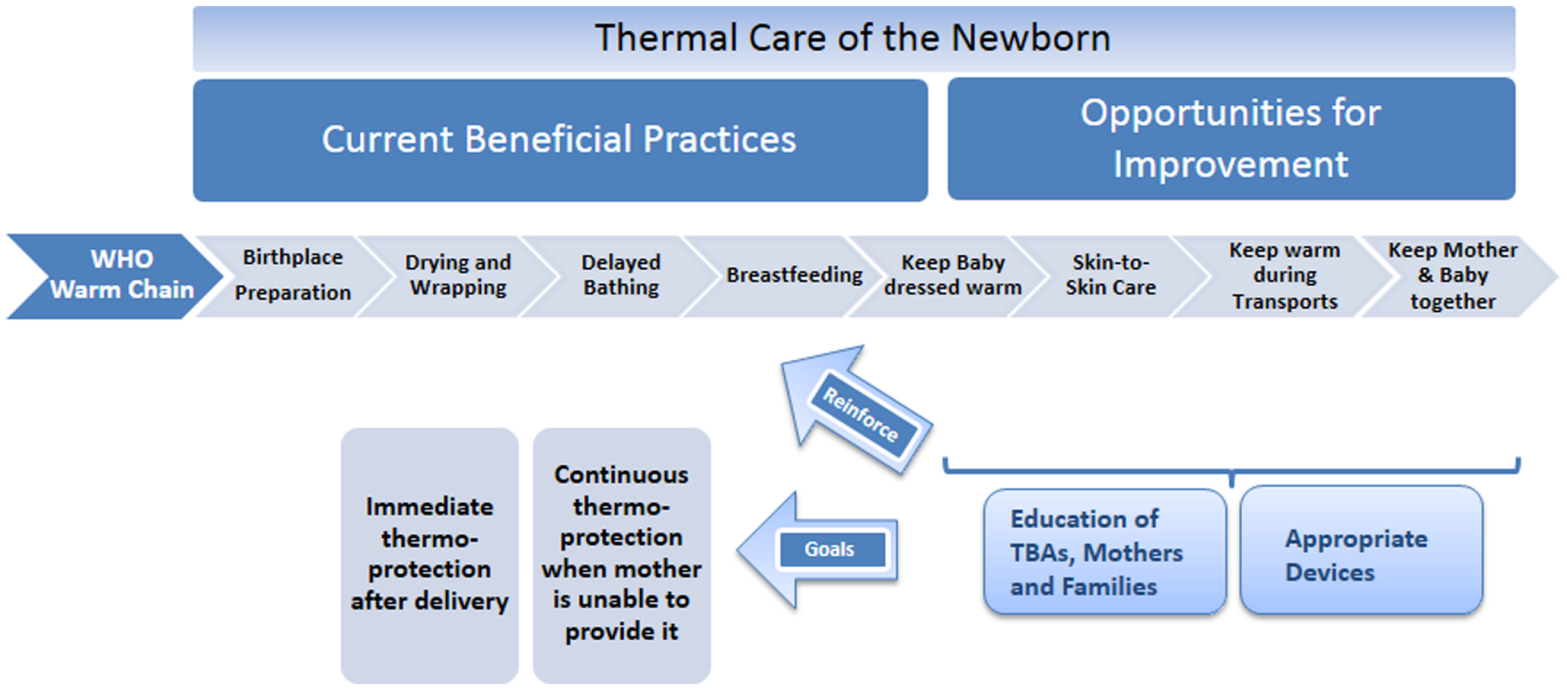
Practices related to thermal care in Lufwanyama, Zambia.

This study identified opportunities to prevent and manage neonatal hypothermia, with potential implications for similar settings in rural Sub-Saharan Africa (Figure 1). In spite of reports of many beneficial thermal care practices, newborn care practices did not conform to the “warm chain” proposed by the WHO. TBAs reported that they usually place mothers on the floor to avoid soiling the bed. Often, a TBA is the sole birth attendant, and immediately postpartum she needs to focus on the mother. Therefore, the newborn is often just dried after birth and wrapped if something to wrap is available, and then put next to the mother or into a corner of the room without receiving attention until the mother is cared for. In a previous prospective, cluster-randomized, controlled effectiveness trial, we showed that a combination of interventions including immediate simple thermal care, i.e. drying and wrapping the baby, together with neonatal resuscitation could be done by trained TBAs and reduced neonatal mortality almost by half (45%) [30]. Educational messages to promote thermal care in rural areas such as Lufwanyama need to reinforce the importance of immediate thermal care after birth and need to address various potential delays.

This study indicates that delays in drying and wrapping the infant persist. Educational messages should reinforce hazards from early heat loss and aim at early thermal care of the newborn, even before the cord is cut. Likewise, while breastfeeding is commonly practiced, early (and exclusive) breastfeeding should be propagated both to facilitate taking advantage of the mother as active heat source for the infant, and to prevent hypoglycemia, which initiates a vicious circle of depleting energy sources and increasingly insufficient heat generation [7].

Skin-to-skin care (SSC) was not a reported practice in the study area. Continuous thermal care beyond the early period after delivery is often assumed to be beneficial, e.g., for premature infants or those born small for gestational age. In Lufwanyama, women traditionally carry their infants on the back, in a chitenge formed as a baby sling. Several studies conducted in various settings such as Uganda [31], Ghana [23] and India [32] suggested that in the absence of health facilities prepared to deliver essential newborn care, community members would accept the implementation of thermoprotective practices such as skin-to-skin care. Further formative study in Lufwanyama would be necessary to explore the acceptability of skin-to-skin care on the chest, to promote breastfeeding heat transmission from the mother; or alternatively to test the thermoprotective effect and safety of providing skin-to-skin care on the back if practical and culturally preferred by mothers.

A mother's female family members are actively involved in newborn care in Lufwanyama, when available. Training them to support her with thermal care for her newborn might include skin-to-skin care by caretakers other than the mother. Mothers often need to resume their work responsibilities soon after delivery. In these cases, a complementary strategy to skin-to-skin care might be the use of culturally appropriate, low-cost newborn warmers designed for resource-limited environments to prevent and manage newborn hypothermia [33], such as the Embrace device currently marketed in India [34]. A recent clinical RCT conducted at the University Teaching Hospital in Lusaka, Zambia, demonstrated that preterm and low birth weight infants placed inside a simple, nonmedical polyethylene bag (costing 3 cents per bag) experienced less hypothermia than those with standard thermoregulation care (wrapping with blankets and placed either under a radiant warmer or in an open crib) [35]. Innovative, low-cost devices might particularly prove useful for premature infants, who often have a prolonged time of need for active thermal care [36].

### Limitations and further research needed

A discrepancy remains between community members' reported awareness of beneficial practices and the reality that neonatal outcomes in the region studied remain poor. This study focused on neonatal hypothermia; other major underlying factors of newborn care such as resuscitation and clean delivery practices need to be taken into account to explain poor newborn survival and devise optimal strategies and programs to improve newborn survival. The study is based on respondents' narratives, limiting our ability to quantify any practice. Participant observation could further elucidate how recommended practices are implemented, and how current practices could be optimized. Given the indication that trained TBAs have had a beneficial influence on community members in the recent past, an important question to be addressed might be the potential to strengthen the role of TBAs and other community health cadres in newborn care.

## Conclusions

Understanding and addressing community-based practices on hypothermia prevention and management might help improve newborn survival in resource-limited settings. In rural Zambia, possible interventions include the implementation of skin-to-skin care (as currently piloted in Lufwanyama by Save the Children and MCHIP-Maternal and Child Health Integrated Program), training family members to support mothers with thermoprotection, and appropriate, low-cost newborn warmers to prevent and manage hypothermia of infants whose mothers need to return to work soon after delivery. These interventions, once tested in rigorous evaluations based on randomized, controlled trials, have the potential to prevent early newborn deaths and thus save newborn lives in resource-limited settings such as rural Zambia.
